# Chromosome-level genome assembly of marine diatom *Skeletonema tropicum*

**DOI:** 10.1038/s41597-024-03238-8

**Published:** 2024-04-20

**Authors:** Shuya Liu, Nansheng Chen

**Affiliations:** 1 CAS Key Laboratory of Marine Ecology and Environmental Sciences, Institute of Oceanology, Chinese Academy of Sciences, Qingdao, 266071 China; 2 Laboratory for Marine Ecology and Environmental Science, Qingdao Marine Science and Technology Center, Qingdao, 266200 China; 3 Center for Ocean Mega-Science, Chinese Academy of Sciences, Qingdao, 266071 China; 4 Department of Molecular Biology and Biochemistry, Simon Fraser University, 8888 University Drive, Burnaby, British Columbia V5A 1S6 Canada

**Keywords:** Adaptive radiation, Molecular ecology, Biogeography

## Abstract

*Skeletonema tropicum* is a marine diatom of the genus *Skeletonema* that also includes many well-known species including *S. marinoi*. *S. tropicum* is a high temperature preferring species thriving in tropical ocean regions or temperate ocean regions during summer-autumn. However, mechanisms of ecological adaptation of *S. tropicum* remain poorly understood due partially to the lack of a high-quality whole genome assembly. Here, we report the first high-quality chromosome-scale genome assembly for *S. tropicum*, using cutting-edge technologies including PacBio single molecular sequencing and high-throughput chromatin conformation capture. The assembled genome has a size of 78.78 Mb with a scaffold N50 of 3.17 Mb, anchored to 23 pseudo-chromosomes. In total, 20,613 protein-coding genes were predicted, of which 17,757 (86.14%) genes were functionally annotated. Collinearity analysis of the genomes of *S. tropicum* and *S. marinoi* revealed that these two genomes were highly homologous. This chromosome-level genome assembly of *S. tropicum* provides a valuable genomic platform for comparative analysis of mechanisms of ecological adaption.

## Background & Summary

Diatoms (i.e. Bacillariophyta) are unicellular algae with silicified cell walls that represent one of the most ecologically important phytoplankton groups^1,2^. Diatoms were estimated to contribute approximately 20% of global primary production on Earth, and up to 40% of marine primary production^3^. Diatoms are also considered as the most species-rich class of microalgae, with estimates range from 12,000 to 30,000 species^4–6^. To date, genomes of only a handful of diatom species have been constructed chromosome-level assemblies, including *Thalassiosira pseudonana*^7^, *Phaeodactylum tricornutum*^8^, *Fistulifera solaris*^9,10^ and *Skeletonema marinoi*^11^. These limited number of high-quality genome assemblies severely hinders in-depth research on the internal phylogeny and evolutionary adaption of diatoms.

*Skeletonema* is one of the most common diatom genera that dominates most coastal waters, some species of which often form harmful algae blooms (HABs)^12–15^. Of the *Skeletonema* species, S. *marinoi* is the most dominant phytoplankton species that populates in the colder water (in high-latitude ocean regions and temperate ocean regions during winter-spring seasons)^12,16^. Interestingly, *S. tropicum* of the genus *Skeletonema* has a dramatically different preference to temperature, which appears in tropical ocean regions and summer-autumn seasons in temperate ocean regions^12,16,17^. Despite of the ecological importance of *Skeletonema* species, genomic information of the *Skeletonema* species is rather limited. To date, organelle genomes of some *Skeletonema* species have been constructed, including mitochondrial genomes (mtDNAs)^18^, and chloroplast genomes (cpDNAs)^19^ of five *Skeletonema* species *S. marinoi*, *S. tropicum*, *S. grevillei*, *S. pseudocostatum* and *S. costatum*. The conserved genetic structures of these organelle genomes among *Skeletonema* species couldn’t explain their mechanisms of ecological adaptation. The chromosome-level genome assembly of the first *Skeletonema* species, *S. marinoi* was recently constructed^11^. The availability of this genome assembly led to the discovery of a substantial expansion of light harvesting genes and photoreceptor gene families, which might help the ecological adaptation of *S. marinoi* under low light condition during the winter-spring seasons. While the whole genome of *S. tropicum* was still lacking, hampering the comparative genomics analysis among the *Skeletonema* species.

**Fig. 1:**
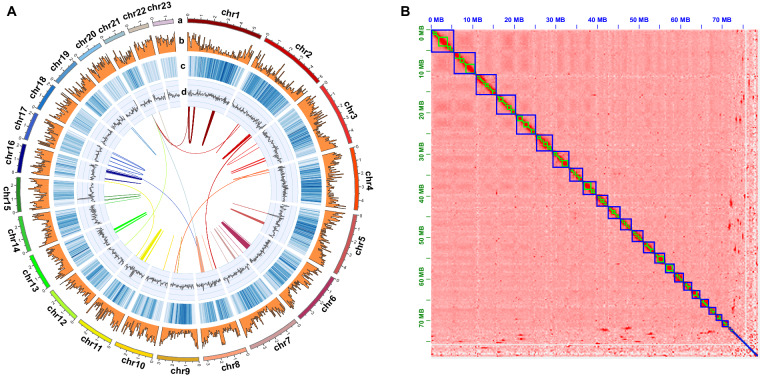
Construction of the first chromosome-level genome assembly of *S. tropicum*. (**A**). Circos plot of the *S. tropicum* genome assembly. From outer to inner layers were chromosomes (a), repetitive elements (b), gene densities (c), GC contents (d), respectively. The inner most part layer was the collinear gene pair blocks. (**B**). Hi-C intra-chromosomal contact map of the genome assembly in *S. tropicum*.

In this study, we report the first chromosome-level genome assembly of the high temperature preferring *Skeletonema* species *S. tropicum* (Fig. 1A). The assembled genome size of *S. tripicum* was 78.69 Mb using PacBio single-molecular DNA sequencing technology^20^, and the contig N50 was 606.27 Kb. To obtain the high-quality genome assembly at the chromosome level, high-throughput chromatin conformation capture (Hi-C)^21^ was used and the contigs were clustered into 23 chromosomes, which corresponds to 91.10% of the total contig length. The final assembled genome size of *S. tropicum* was 78.78 Mb with the scaffold N50 length of 3.17 Mb. A total set of 20,613 putative protein-coding genes (PCGs) were predicted in *S. tropicum*, among which, 86.14% were annotated to the publicly available database. These chromosome-level genome assemblies of the high temperature preferring *Skeletonema* species *S. tropicum* and the low temperature preferring *Skeletonema* species *S. marinoi* set up a valuable platform for elucidating mechanisms of temperature adaptation for surviving adverse environments.

## Methods

### Strain isolation and genome sequencing

The *S. tropicum* strain (CNS00166) analysed in this study was isolated using single-cell capillary from marine water collected in Jiaozhou Bay, China in October 2019. The CNS00166 strain was purified using sterilized seawater for many times. The CNS00166 strain is kept and available in the Key Laboratory of Marine Ecology and Environmental Science from the Institute of Oceanology, Chinese Academy of Science. The axenic cultivation of this strain was maintained in L1 medium^22^. To ensure low bacterial contamination, penicillin and streptomycin stock solution was added into culture solution. The culture conditions, including culture seawater, temperature, salinity and irradiance intensity, were described previously^18^. The *S. tropicum* cells for sequencing were collected by centrifugation and stored in liquid nitrogen. The mtDNA and cpDNA of *S. tropicum* strain CNS00166 have been reported previously^18,19^.

High-quality and long-fragment DNA (≥40 Kb) library was prepared by extracting DNA using a magnetic-bead based protocol^11^. For genome survey analysis, short reads were obtained using MGI short-reads sequencing. The MGI sequencing library (DNBSEQ) was constructed and sequenced using the MGISEQ-2000-PE150 platform. A total of 40.91 Gb (519X sequencing depth) short reads were obtained in this study for genome survey and genome assembly (Table 1). For chromosome-level genome assembly, PacBio continuous long reads (CLR) sequencing library was constructed and sequenced using PacBio Sequel SMRT Cell 1 M. As a result, 10.04 Gb (127X sequencing depth) of PacBio long reads were obtained (Table 1). The N50 length and maximum length of PacBio sequencing reads were 18.18 Kb and 215.08 Kb, respectively. For the Hi-C analysis, algal samples were processed as previously described^11^ and the Hi-C library was sequenced with MGISEQ-2000-PE150. This process yielded a total of 50.93 Gb of raw data for predicting the spatial proximity of chromatin loci. Three replicates of each RNA sample of *S. tropicum* in the exponential growth were collected by centrifugation. High-quality RNA was extracted using cetyltrimethylammonium bromide (CTAB) methods^11^, followed by RNA quality checking using Agilent 2100 Bioanalyzer and NanoDrop. The short-length and full-length transcriptome libraries were sequencing by MGISEQ-2000-PE150 platform and PacBio Sequel SMRT Cell 1 M, respectively.

**Table 1:** Statistics of *S. tropicum* genome assembly and annotation.

Items	Statistical result
**Sequencing**	
**Short reads sequencing**	
raw data (Gb)	40.91
Sequencing depth (X)	519
**PacBio sequencing**	
Raw data (Gb)	10.04
Sequencing depth (X)	127
**Hi-C sequencing**	
Raw data (Gb)	50.93
**Genome Survey**	
Estimated genome size (Mb)	73.10
Heterozygous Ratio (%)	0.73
Repeat Ratio (%)	48.60
**Assembly features**	
**PacBio sequencing assembly**	
Genome size (Kb)	78.69
Contig N50 (Kb)	606.27
BUSCO completeness of assembly (%)	96.00
**Hi-C assembly**	
Genome size (Mb)	78.78
Chromosome number	23
Anchored rate(%)	91.10
Scaffold N50 (Mb)	3.17
**Genome annotation**	
Number of protein-coding genes	20,613
Average gene length (bp)	1675.09
Average exon per gene	1.74
Average exon length (bp)	750.84
Number of exons	35,908
Average intron length (bp)	254.47
Number of introns	15,295
Total size of TEs (Mb)	38.73
TEs in genome (%)	49.17
BUSCO completeness of annotation (%)	86.00

### Genome survey and genome assembly

The genome survey was conducted based on k-mer distribution using the short-length reads using Jellyfish V2.1.4^23^ with k-mer size = 21 and GenomeScope V1.0^24^. The estimated genome size of *S. tropicum* (CNS00166 strain) was 73.10 Mb with heterozygous ratio was 0.73% and repeat ratio was 48.60% (Table 1).

The PacBio long-read data was used for *de novo* genome assembly by MECAT2^25^, the primary assembled genome was polished by Arrow (https://github.com/PacificBiosciences/GenomicConsensus) using PacBio long reads and by pilon^26^ using short reads. Purge Haplotigs^27^ was used to remove redundancy from the assembled genome. The size of this genome assembly was 78.69 Mb, which was similar to the estimated genome size based on the k-mer analysis. The assembled genome consisted 376 contigs and the N50 was 606.27 Kb. The completeness and quality of this genome assembly was evaluated by BUSCO v5.4.3^28^ against the stramenopiles_odb10 data set. Among the BUSCO orthologous groups, 96.00% were identified as complete in the assembled genome (Table 2).

**Table 2:** Summary of BUSCO analysis of genome assembly and annotation in *S*. *tropicum*.

Type	Assembled genome	Annotation
	Percentage (%)	Percentage (%)
**Complete BUSCOs (C)**	96.00%	86.00%
**Complete and single-copy BUSCOs (S)**	90.00%	82.00%
**Complete and duplicated BUSCOs (D)**	6.00%	4.00%
**Fragmented BUSCOs (F)**	2.00%	4.00%
**Missing BUSCOs (M)**	2.00%	10.00%
**Total BUSCO groups searched**	100.00%	100%

A total of 50.93 Gb Hi-C sequencing raw data was obtained (Table 1), then was conducted quality control by HiC-Pro v2.5.0^29^. The contigs were mapped onto chromosome-level scaffolds by Juicer v1.6^30^ and 3D-DNA^31^. As a result, 23 chromosome-level scaffolds were obtained with an anchored rate was 91.10% (Fig. 1), and the length range was from 1558 Kb to 5738 Kb (Table 3). The anchored rate was a little lower probably due to the high heterozygous ratio and repeat content of *S. tropicum* in this study, the final assembled contigs might contain some highly heterozygosity allelic sequences that are redundant. As only one set of these highly heterozygosity sequences was anchored into the genome assembly with the help of Hi-C data, resulting in relatively lower anchored rate. Finally, the size of genome assembly was 78.78 Mb with the scaffold N50 was 3.17 Mb.

**Table 3:** Statistics of chromosome length in *S. tropicum*.

Chromosome ID	Length (bp)	Percentage(%)
StrChr1	5737774	7.28%
StrChr2	5219912	6.63%
StrChr3	4895999	6.21%
StrChr4	4763068	6.05%
StrChr5	4720718	5.99%
StrChr6	3999239	5.08%
StrChr7	3995353	5.07%
StrChr8	3306349	4.20%
StrChr9	3169793	4.02%
StrChr10	2978062	3.78%
StrChr11	2871570	3.65%
StrChr12	2754620	3.50%
StrChr13	2747261	3.49%
StrChr14	2727425	3.46%
StrChr15	2615174	3.32%
StrChr16	2195411	2.79%
StrChr17	2148979	2.73%
StrChr18	2110432	2.68%
StrChr19	2081782	2.64%
StrChr20	1952568	2.48%
StrChr21	1657245	2.10%
StrChr22	1559287	1.98%
StrChr23	1558474	1.98%
Total	71766495	91.10%
Unplaced	7010717	8.90%

### Genome annotation

The genome annotation steps included three parts: repetitive elements annotation, non-coding RNAs annotation and PCGs annotation. The homolog repetitive elements were predicted by RepeatMasker v4.0.7^32^ and RepeatProteinMask v4.0.7 (http://www.repeatmasker.org/cgibin/RepeatProteinMaskRequest) based on the RepBase v21.12 database^33^. For *de novo*-based repetitive elements, a *de novo* repetitive element database was generated by RepeatScout^34^, Piler^35^ and LTR_FINDER v1.07^36^ at first, then *de novo*-based repetitive elements were predicted by RepeatMasker. Combination of homology-based and *de novo*-based approaches, a total of 38.73 Mb of transposable elements (TEs) were obtained, contributing 49.17% of assembled genome (Table 4). The DNA, LINE, SINE and LTR account for 5.39%, 5.18%, 0.065% and 25.26% of genome, respectively. In addition, tandem repeats were annotated by Tandem Repeats Finder (TRF v4.09)^37^, and a total of 6.08 Mb of tandem repeats were obtained accounting for 7.72% of total genome.

**Table 4:** Statistics of transposable elements (TEs) in *S. tropicum*.

	RepBase TEs		TE Proteins		*De novo*		Combined TEs	
	Length (bp)	%in Genome	Length (bp)	%in Genome	Length (bp)	%in Genome	Length (bp)	%in Genome
**DNA**	367,820	0.467	51,758	0.066	3,897,528	4.948	4,244,654	5.388
**LINE**	377,550	0.479	830,272	1.054	3,318,704	4.213	4,076,881	5.175
**SINE**	42,987	0.055	0	0	8252	0.01	51,059	0.065
**LTR**	861,109	1.093	1,051,666	1.335	19,531,524	24.793	19,897,941	25.258
**Other**	513	0.001	0	0	0	0	513	0.001
**Unknown**	0	0	0	0	13,347,425	16.943	13,347,425	16.943
**Total**	1,511,998	1.919	1,933,304	2.454	38,251,221	48.556	38,731,992	49.166

Non-coding RNAs are annotated divided into several types, including tRNA, rRNA, snRNA and miRNA. The tRNAs were predicted through tRNAscan-SE^38^. The rRNA were annotated by Blast v2.2.31^39^ using the reference sequences of *S. marinoi*. The snRNAs and miRNAs were identified through INFERNAL in RFAM^40^.

**Fig. 2:**
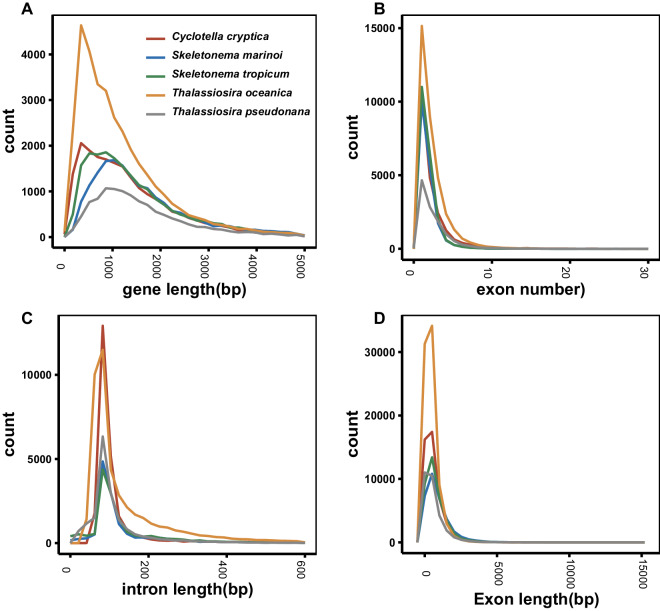
The composition of gene elements in the *S. tropicum* and other closely related species. (**A**) Distribution of gene length. (**B**) Distribution of exon number. (**C**) Distribution of intron length. (**D**) Distribution of exon length.

The PCGs were annotated through integrated approaches, including *de novo*-, homology- and transcriptome-based information. The *de novo* prediction were conducted using AUGUSTUS^41^ and SNAP^42^, and yielded 24,008 and 31,109 genes, respectively. For the homology-based prediction, the PCG sequences of closely related or model species, including *S. marinoi*^11^, *T. pseudonana*^7^, *Fragilariopsis cylindrus*^43^, *Seminavis robusta*^44^, *P. tricornutum*^8^ and *Arabidopsis thaliana*^45^, were aligned against the *S. tropicum* genome using Blast v2.2.31, then the gene structures were predicted from these alignments by Exonerate v2.2.0^46^. A total of 84,803 homologous genes were obtained. For the transcriptomic prediction, the RNA-Seq short-read data were aligned to the assembled genome through HISAT2 v 2.1.0^47^ and then assembled and corrected by StringTie v1.3.4^48^ and Pasa_lite (https://github.com/PASApipeline/PASA_Lite). Iso-Seq long-read data were used to get full-length non-chimeric reads by the SMRT Analysis System. A total of 334,554 genes were predicted by the RNA-Seq and Iso-Seq, which contained some redundancy. Finally, gene models from these strategies were merged to form a consensus gene set using MAKER2^49^, and 20,613 PCGs were predicted, with an average gene length of 1675.09 bp and exon length of 750.84 bp (Table 1). The statistics of gene models, including gene length, intron length, exon number and exon length in *S*. *tropicum* were comparable to *S. marinoi* (Fig. 2).

**Fig. 3:**
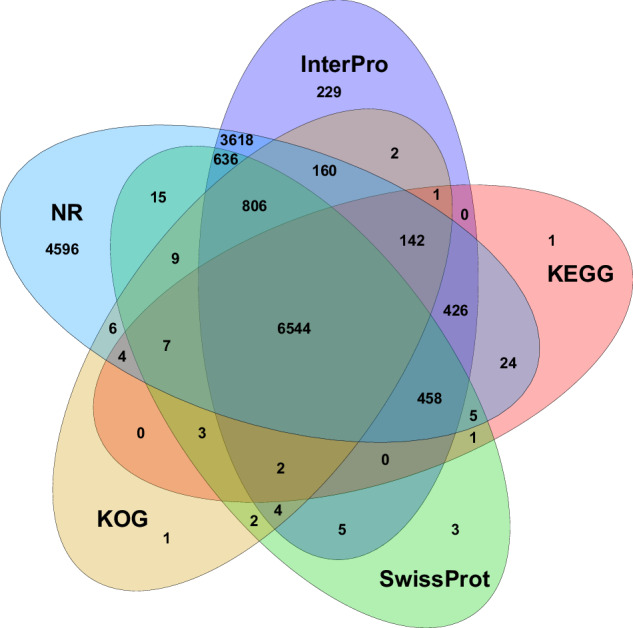
The venn diagram of PCG annotation of *S. tropicum* to five databases: NR, InterPro, KEGG, SwissProt and KOG.

For the functional prediction, these PCGs were annotated to the public databases, including GenBank Nr, SwissProt, Kyoto Encyclopedia of Genes and Genomes (KEGG), eukaryotic orthologous groups (KOG), TrEMBL, InterPro and gene ontology (GO), through Blast v2.2.31 with e-value less than 1e-5. Among all the PCGs, 17,757 genes (86.14%) were functionally annotated to at least one database, and 6544 genes (31.74%) were annotated to at least five databases (Table 5, Fig. 3).

**Table 5:** The Gene function annotation statistics in *S. tropicum*.

Values	Total	Nr	Swissprot	KEGG	KOG	TrEMBL	Interpro	GO	Overall
**Number**	20613	17456	8500	7618	7693	17408	13033	8043	17757
**Percentage**	**—**	84.68%	41.24%	36.96%	37.32%	84.45%	63.23%	39.02%	86.14%

## Data Records

The genome sequencing data (including DNA short-reads sequencing data, DNA PacBio long-reads sequencing data, Hi-C sequencing data, RNA short-reads sequencing data and RNA PacBio long-reads sequencing data) are deposited in the NCBI SRA database under the accession numbers: SRR26857256^50^, SRR26857255^50^, SRR28393139^51^, SRR26857253^50^, and SRR26857252^50^. The genomic assembly and annotation results were available at the *figshare* database^52^. The genome assembly has also been deposited to NCBI under the accession number of JAWZXG000000000^53^.

## Technical Validation

### Low contamination ratio of Bacteria

**Fig. 4:**
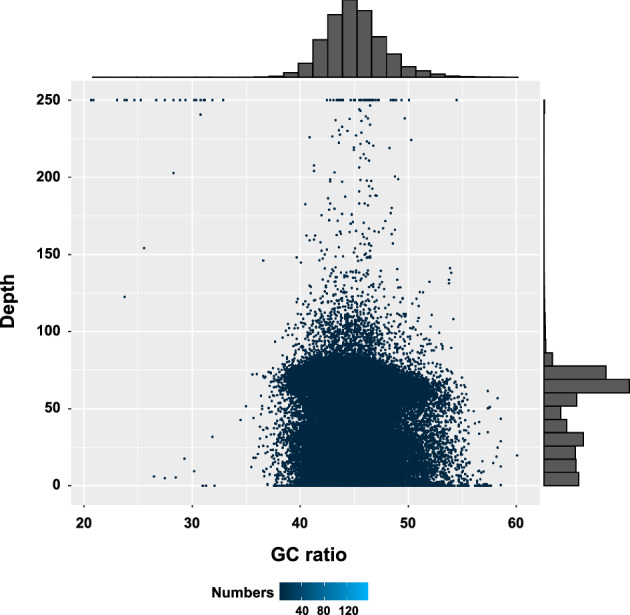
The distribution of GC ratio and sequencing depth. Histograms on the top and right show the frequency distribution of GC ratio and sequencing depth, respectively.

The low bacteria contamination in the axenic culture of diatom was the critical factor for the high-quality genome assembly. To check low bacteria contamination, 1 Mb of clean short-reads data were selected randomly, and blasted to NCBI NT database. The result showed that the bacteria contamination of *S. tropicum* was as low as 0.26% (Table 6). The top 20 species of reads annotated to NT database included the *Skeletonema* species and other closely species, indicating the absence of bacteria comtamination in this project (Table 7). In addition, the short-read DNA data were mapped to the PacBio assembled genome using BWA v. 0.7.10^54^ to evaluate the GC contents and sequencing depth with 1 Kb window length statistics (Fig. 4**)**, the results showed that the almost all GC points located at the 45%, indicating no exogenous species pollution was found. In addition, the sequencing depth of many points was close to 0, which probably due to its high repeat contents of *S. tropicum* genome. The reads of repeat content were usually matched to multiple locations of genome assembly in the BWA alignment, resulting in the filtration of the score. Thus, the sequence depth of some locations appeared to 0. The results altogether suggested that genome assembly of *S. tropicum* was not contaminated by bacteria or other species.

**Table 6:** Statistics of clean reads of short DNA sequences annotated to NT database.

	Number	Percentage
The number of total reads	1,000,000	100.00%
The number of annotated reads	46,306	4.63%
The number of reads annotated to subdatabase	32,132	3.21%
The number of reads annotated to Plants subdatabase	29,133	2.91%
The number of reads annotated to Bacteria subdatabase	2586	0.26%

**Table 7:** Top 20 species of reads annotated to NT database (length > = 100 bp).

Order	Species	The number of annotated reads	Subdatabase
1	*Thalassiosira pseudonana*	23595	*Plants*
2	*Skeletonema costatum*	15839	*Plants*
3	*Thalassiosira weissflogii*	15359	*Plants*
4	*Cyclotella sp*.	15207	*Plants*
5	*Thalassiosira oceanica*	13038	*Plants*
6	*Roundia cardiophora*	12208	*Plants*
7	*Skeletonema marinoi*	9755	*Plants*
8	*Skeletonema pseudocostatum*	8553	*Plants*
9	*Skeletonema grethae*	8015	*Plants*
10	*Skeletonema japonicum*	7894	*Plants*
11	*Skeletonema tropicum*	7569	*Plants*
12	*Skeletonema menzellii*	6913	*Plants*
13	Uncultured marine eukaryote	6863	*Environmental samples*
14	*Lithodesmium undulatum*	6609	*Plants*
15	*Skeletonema potamos*	6540	*Plants*
16	*Cylindrotheca closterium*	6465	*Plants*
17	*Phaeodactylum tricornutum*	6112	*Plants*
18	*Skeletonema dohrnii*	6089	*Plants*
19	*Asterionellopsis glacialis*	6070	*Plants*
20	Uncultured eukaryote	5900	*Environmental samples*

### Evaluating genome assembly and annotation completeness

**Fig. 5:**
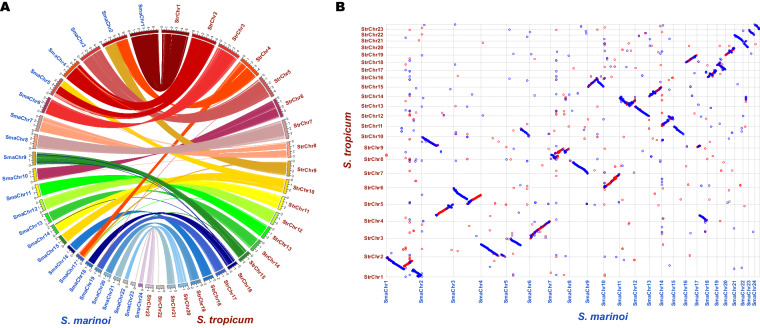
Collinearity analysis between *S. tropicum* and *S. marinoi* in the view of amino acid sequences (**A**) and DNA sequences (**B**).

In this study, a total of 519X and 127X of MGI short reads and PacBio reads were used, respectively, which could ensure the quality in the genome assembly. The quality assessments of the genome assembly and annotation were evaluated by BUSCO analysis (Table 2). The results showed that 96.00% and 86.00% were identified as complete orthologs for genome assembly and PCGs annotations, respectively, indicating the high quality of this genome. Although the high heterozygous ratio and repeat content, a high quality genome assembly was obtained in this study. The Hi-C heatmap shows a well-organized interaction pattern within the chromosomal region (Fig. 1), and assembly resulted in 23 chromosome-level scaffolds. Collinearity analysis of amino acid sequences of PCGs between *S. tropicum* and the same genus species *S. marinoi* was conducted (Fig. 5A) through Blast v2.2.31 with the evalue less than 1e-05 to identify homologous PCGs, then followed analysed and visualized by WGDI^55^ and Circos^56^. The collinearity analysis of DNA sequence (Fig. 5B) was also conducted using mummer 3.0^57^ with minimum alignment length of 1000 bp and many-to-many alignment allowing for rearrangements. The results showed that almost all chromosomes of *S. tropicum* displayed high homology with the chromosomes of *S. marinoi*. The clearly strong collinearity between the two close phylogenetic species indicated high quality sequencing and assembly of *S. tropicum*. Taken together, these confidently confirm the accuracy of the genome assembly and annotation.

## Data Availability

No custom code was used in this study. The data analyses used standard bioinformatic tools specified in the methods.
